# Hyperinsulinism Hyperammonemia Syndrome, a Rare Clinical Constellation

**DOI:** 10.1177/2324709616632552

**Published:** 2016-02-11

**Authors:** Jonathan Hussain, Alexander Schlachterman, Amir Kamel, Anand Gupte

**Affiliations:** 1 University of Florida, Gainesville, FL, USA

**Keywords:** hyperinsulinism, hyperammonemia, gastroparesis, hypoglycemia

## Abstract

We present the unique case of adult hyperinsulinism hyperammonemia syndrome (HI/HA). This condition is rarely seen in children and even more infrequently in adults. A 27-year-old female with HI/HA, generalized tonic-clonic seizures, staring spells, and gastroesophageal reflux disease presented with diffuse abdominal pain, hypoglycemia, confusion, and sweating. She reported a history of significant nausea, vomiting, and diarrhea, which had been present intermittently over the past year. On examination, she was found to have a soft, nontender, and mildly distended abdomen without splenomegaly or masses. She had a normal blood pressure and was tachycardic (130 bpm). Her initial complete blood count and basic metabolic panel, excluding glucose, were within normal limits. She was found to have an elevated peripherally drawn venous ammonia (171 mmol/L) and near hypoglycemia (blood glucose 61 mg/dL), which were drawn given her history of HI/HA. She was continued on home carglumic acid and diazoxide, glucose was supplemented intravenously, and she was started on levetiracetam for seizure prophylaxis. An upper endoscopy (esophagogastroduodenoscopy [EGD]) was performed and was unremarkable, and biopsies taken were within normal limits. Following the EGD, she underwent a gastric emptying study that showed delayed emptying (216 minutes), consistent with a new diagnosis of gastroparesis, the likely etiology of her initial abdominal pain on presentation. This was subsequently treated with azithromycin oral solution. We present this case to raise awareness of this rarely encountered syndrome and to provide the basic principles of treatment.

## Introduction

Hyperinsulinism hyperammonemia syndrome (HI/HA) is a congenital condition that is first recognized in children at the age of 6 to 12 months. Our case is an adult with previously diagnosed HI/HA, the treatment of which can be quite complicated without an understanding of the underlying disease. We have detailed the management that we employed. Our goal is to provide information to aid in the recognition of HI/HA and to avoid unnecessary interventions in this population.

## Case Presentation

A 27-year-old, ill-appearing, obese (body mass index 35) female with a past medical history of HI/HA, generalized tonic-clonic seizures, staring spells, and gastroesophageal reflux disease presented with diffuse abdominal pain, hypoglycemia, confusion, and sweating. She was initially diagnosed with HI/HA syndrome at 3 months of age when she experienced a tonic-clonic seizure. The remaining details of her diagnosis were unknown to the patient or her accompanying mother. Her family history included type 2 diabetes mellitus in her father and multiple sclerosis in her mother. No family members were known to have HI/HA. She reported a lifelong history of epilepsy characterized by generalized tonic-clonic seizures as well as staring spells. During previous tonic-clonic seizures and staring spells, the patient has had her blood glucose drawn. She has been found to be hypoglycemic during the majority of these events, but has occasionally been found to be in the normal blood glucose range. Her home medications included carglumic acid, diazoxide, lactulose, and omeprazole. In addition to her chronic seizures and abdominal pain, she noted significant nausea, vomiting, and diarrhea, which had been present over the past year. On examination, she was found to have a soft, nontender, and mildly distended abdomen without splenomegaly or masses. She had a normal blood pressure and was tachycardic (130 bpm). Her initial complete blood count and basic metabolic panel, excluding glucose, were within normal limits. She was found to have elevated peripherally drawn venous ammonia (171 mmol/L) and near hypoglycemia (blood glucose 61 mg/dL). No insulin levels or C-peptide were drawn given the preexisting HI/HA diagnosis.

On admission, our patient was continued on her home doses of carglumic acid and diazoxide. She was started on intravenous glucose for hypoglycemia and levetiracetam for seizure prophylaxis. The patient had not been compliant with anti-epileptics at home in the months prior to admission, though the reason behind this is unclear. An upper endoscopy (esophagogastroduodenoscopy [EGD]) was performed and was found to be unremarkable, and biopsies taken were within normal limits. Following the EGD, she underwent a gastric emptying study that showed delayed emptying (216 minutes), consistent with a new diagnosis of gastroparesis, which was subsequently treated with azithromycin. She experienced no seizure activity during her hospitalization.

## Follow-Up

The patient tolerated her procedures well, and has since followed-up with our Gastrointestinal Clinic for a posthospital visit. At that time a repeat peripheral venous ammonia level remained elevated (182 mmol/L). It remains unclear why the patient has developed gastroparesis as this is not a commonly identified issue in patients with HI/HA. Unfortunately, this could not be further explored as the patient has failed to show for additional clinic appointments or have further laboratory workup drawn (including a hemoglobin A1c). Multiple unsuccessful attempts have been made to reach the patient by phone and mail.

## Discussion

Hyperinsulinism hyperammonemia syndrome is caused by a heterozygous missense amino acid substitution on the GLUD1 gene that results in an increase in glutamate dehydrogenase activity by impairing allosteric inhibition by guanosine triphosphate (GTP).^1^ GDH activity occurs exclusively in the pancreatic islets, liver, kidney, and brain.^2^ Increased GDH activity is responsible for increased insulin secretion as well as increased renal ammoniagenesis.^3^ Approximately 70% of the HI/HA mutation occur de novo, whereas the other 30% occurs in an autosomal dominant fashion. Patients with HI/HA exhibit increased sensitivity to protein (leucine) stimulation of insulin release, leading to protein-induced hypoglycemia. They do not have increased insulin release in response to glucose.^2^

The resulting clinical syndrome is characterized by the namesake abnormalities of hyperinsulinism and hyperammonemia, as well as resultant hypoglycemia (both fasting and protein sensitive) and increased risk of generalized epilepsy, mental retardation, and behavior disorders. The disease is present from birth; however, it is often unrecognized until 4 to 12 months of age. Interestingly, despite having elevated ammonia levels, these patients are often without common symptoms of elevated ammonia such as headaches and encephalopathy.^2^ The etiology of epilepsy in HI/HA is not completely clear at this time; it is postulated that overactivity of brain GDH depletes glutamate and glutamine, which causes a disequilibrium of glutamate and GABA. The elevated GABA levels contribute to seizure activity. It has also been suggested that underlying mechanisms may include damage to the developing brain by hyperammonemia.^4^

Management of HI/HA involves seizure prevention, reduction of iatrogenic contributors, and glucose control. There is minimal ammonia-reducing interventions as the hyperammonemia is largely acutely asymptomatic, though it likely contributes to the chronic seizure condition.^2,4^ Glucose control involves replacement via intravenous glucose as well as diazoxide (inhibitor of insulin release), dosed at ~10 mg/kg/day.^2,5^ Of note, a recent study found that green tea polyphenols can modulate insulin secretion by inhibiting GDH^6^; however, this was not employed during her hospital stay. Treatments for hyperammonemia such as dietary protein limitation, arginine, benzoate, carbamoyl synthase activators (carglumic acid), and *N*-carbamoylglutamate have not been effective in reducing ammonia levels.^2,7^

There are relatively few descriptions of HI/HA in adult patients available in literature at this time. This case details the basics of management of adult HI/HA and demonstrates a rare comorbidity to be considered in new patients with hypoglycemia and hyperammonemia.
